# Digestive tract reconstruction after laparoscopic proximal gastrectomy: Double tract reconstruction or double flap technique?

**DOI:** 10.1002/ags3.12857

**Published:** 2024-09-01

**Authors:** Lindi Cai, Guanglin Qiu, Mengke Zhu, Shangning Han, Pengwei Zhao, Panxing Wang, Xiaowen Li, Xinhua Liao, Xiangming Che, Lin Fan

**Affiliations:** ^1^ Department of General Surgery The First Affiliated Hospital of Xi'an Jiaotong University Xi'an China; ^2^ Department of Pathology The First Affiliated Hospital of Xi'an Jiaotong University Xi'an China; ^3^ Department of General Surgery The Second Affiliated Hospital of Xi'an Jiaotong University Xi'an China

**Keywords:** double flap technique, double tract reconstruction, laparoscopic proximal gastrectomy, proximal early gastric cancer

## Abstract

**Aim:**

The reconstruction methods after proximal gastrectomy (PG) are varied but not standardized. This study was performed to evaluate the short‐term clinical outcomes between double tract reconstruction (DTR) and double flap technique (DFT).

**Methods:**

We retrospectively reviewed and collected data of patients who underwent DTR and DFT after laparoscopic proximal gastrectomy (LPG), respectively, between January 2020 and March 2023. Propensity score matching (PSM) was used to balance the baseline data of the two groups, then we compared their short‐term clinical outcomes.

**Results:**

A total of 72 patients (48 and 24 patients in the DTR and DFT groups, respectively) were included. The anastomosis time was significantly longer in the DFT group than that in the DTR group (70.1 vs. 52.7 min, *p* < 0.001). DFT was associated with shorter times of gas‐passing, start of diet, and postoperative length of hospital stay (*p* < 0.001). There were no significant differences between the two groups in terms of early and late postoperative complications (*p* = 0.710, *p* = 1.000, respectively). DFT was superior to DTR in maintaining body weight (*p* < 0.001), total protein (*p* = 0.011) and albumin levels (*p* = 0.018). As for QOL, DTR showed better results in the meal‐related distress subscale (*p* < 0.001). However, DFT was superior to DTR in terms of reducing diarrhea, constipation, and dumping related symptoms (*p* < 0.05).

**Conclusion:**

Double flap technique emerged as a superior alternative to DTR in terms of facilitating early postoperative recovery, sustaining nutritional status, and improving QOL. DFT could potentially be the preferred reconstruction method following laparoscopic proximal gastrectomy.

## Graphical abstract

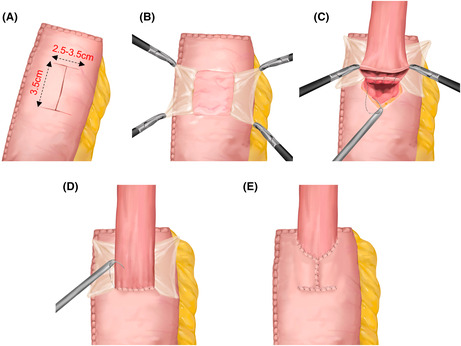

## 1 INTRODUCTION

The incidence and mortality of gastric cancer tend to decline, but the incidence of proximal gastric cancer (PGC) continues to increase worldwide, especially in Western Europe and East Asia.^1^, ^2^, ^3^ PGC is usually defined as a tumor located in the cardia and upper third of the stomach, and total gastrectomy (TG) plus D2 lymphadenectomy is the standard treatment strategy with confirmed efficacy in terms of tumor radicality.^4^, ^5^ However, there is a complete loss of gastric function when TG is performed, which further leads to postoperative malnutrition.^6^ As a function‐preserving surgical procedure, proximal gastrectomy (PG) has been confirmed to be comparable to TG in terms of oncologic safety and feasibility, and PG can be effective at maintaining postoperative weight and improving quality of life (QOL).^7^, ^8^, ^9^

Considering that PG destroys the normal antireflux barrier of the cardia, conventional esophagogastrostomy seems to no longer be popular, and many modified procedures, including gastric tube reconstruction,^10^ jejunal interposition,^11^ double tract reconstruction (DTR),^12^ and the double flap technique (DFT),^13^ have been developed to prevent reflux esophagitis after surgery. However, choosing an appropriate reconstruction method for reducing regurgitation satisfactorily remains controversial. Among these methods, DTR reduces reflux symptoms by constructing two digestive pathways of food, and its surgical procedure is relatively complicated. In contrast to DTR, in DFT, H‐shaped double seromuscular flaps are created and cover the lower esophagus and anastomosis as a one‐way valve to prevent reflux esophagitis. However, the risk of postoperative anastomotic stenosis after DFT cannot be ignored.

Proximal gastrectomy with DTR or DFT has already shown excellent outcomes in terms of postoperative survival and complications, as well as improved postoperative nutritional status compared to that after TG.^14^, ^15^ However, the superiority of DTR over DFT remains debatable. Some studies have reported the short‐term outcomes of these two reconstruction methods and have concluded that DFT is superior to DTR^16^, ^17^; however, the relevant clinical evidence is still insufficient. Therefore, we aimed to clarify the efficacy of these reconstruction methods for treating proximal gastric cancer by assessing surgical outcomes, postoperative complications, nutritional status, and patient QOL.

## 2 MATERIALS AND METHODS

### 2.1 Patients

**FIGURE 1:**
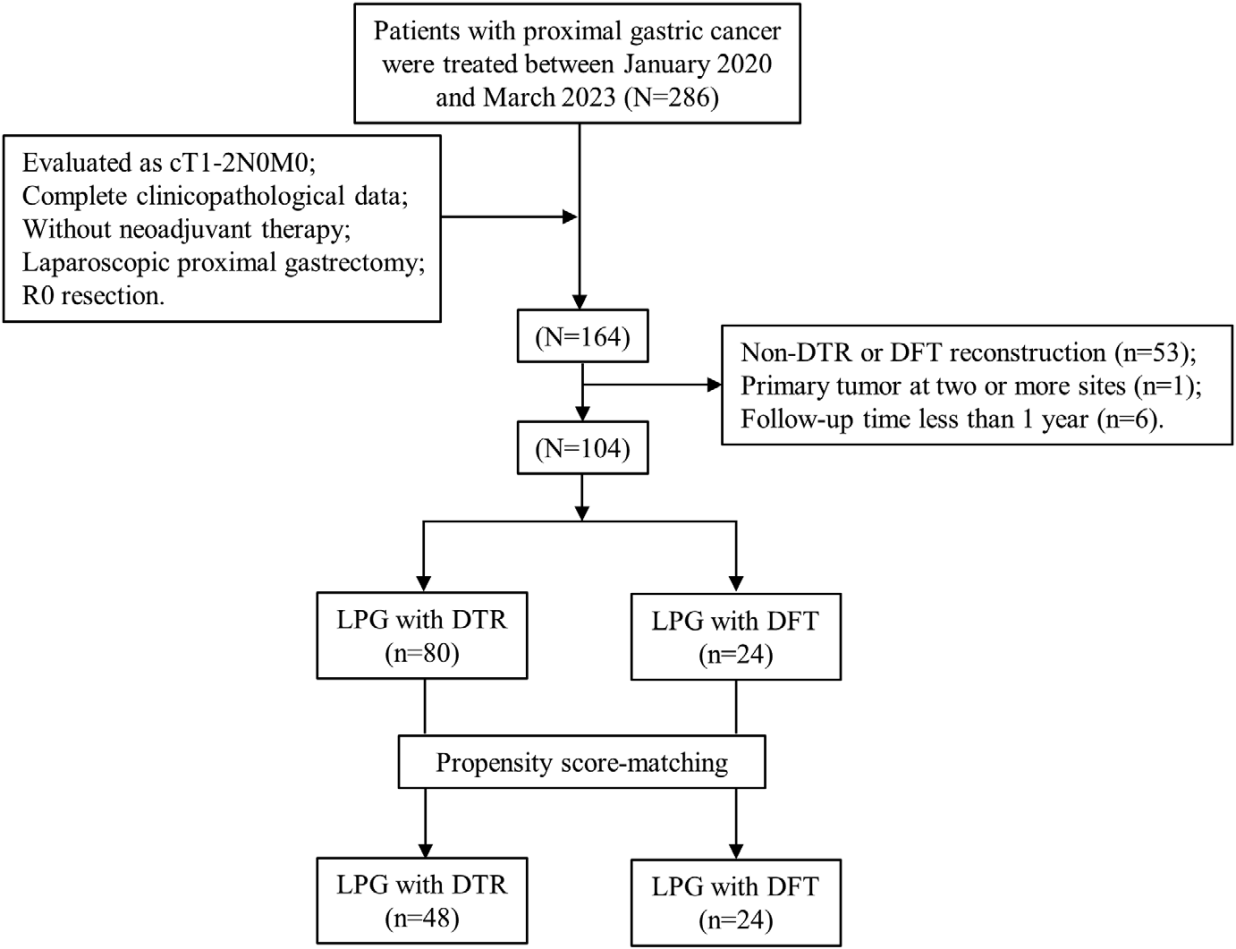
Flow chart of patient selection and propensity score matching. Ultimately, we enrolled 72 patients who underwent laparoscopic proximal gastrectomy (LPG) with double‐tract reconstruction (*n* = 48) and LPG with double flap technique (*n* = 24).

A total of 286 patients with proximal gastric cancer treated at the First Affiliated Hospital of Xi'an Jiaotong University between January 2020 and March 2023 were initially enrolled in this study. The detailed screening process is shown in Figure 1. Inclusion criteria: (1) proximal gastric cancer evaluated as cT1‐2N0M0; (2) complete clinicopathological data; (3) no neoadjuvant therapy; (4) laparoscopic proximal gastrectomy (LPG); and (5) R0 resection. Exclusion criteria: (1) non‐DTR or DFT reconstruction; (2) primary tumor at two or more sites; (3) follow‐up time less than 1 year.

The included patients were divided into the DTR group (*n* = 80) and the DFT group (*n* = 24). Subsequently, propensity score matching (PSM) analysis was conducted to balance background characteristics according to the following factors: age, sex, body mass index (BMI), American Society of Anesthesiologists physical status score (ASA‐PS), tumor size, and pathological stage. Finally, 48 patients who underwent LPG with DTR and 24 patients who underwent LPG with DFT were included in this study. This retrospective cohort study was approved by the Ethics Committee of the First Affiliated Hospital of Xi'an Jiaotong University. Due to the retrospective design of this study, the requirement for informed consent was waived. Regarding the use of data in the retrospective study, patients were allowed to opt out of the study at any time.

### 2.2 Surgical procedure

All patients were evaluated preoperatively for cT1‐2N0M0 proximal gastric cancer, and LPG plus D1+ lymphadenectomy was performed according to the Japanese Gastric Cancer Treatment Guidelines (5th) to ensure that at least 1/2 of the stomach volume was preserved.^18^ The team's chief surgeons, Lin Fan and Xiangming Che, possessed over 20 years of clinical expertise and had accumulated experience in performing over 1000 laparoscopic procedures. They were also involved as a quality control, and they supervised the key stages of this study. We completely exposed the lower abdominal esophagus through the esophageal hiatus with a harmonic scalpel and a linear stapler was used to transect the esophagus 3 cm from the tumor. DFT was preferred when the volume of the remnant stomach was close to 2/3 of the stomach. Nevertheless, when the tumor invades the esophagus by over 2 cm, necessitating resection of an extended abdominal segment of the esophagus to achieve negative resection margins, and considering the complexity of intra‐mediastinal anastomosis between the esophagus and residual stomach, DTR becomes the preferred option. When DFT was performed, four extra marked points should be carried out at the posterior wall of the esophagus 5 cm from the resected end because contraction of the esophagus is usually inevitable after resection. After that, the stomach was extracted through an approximately 5 cm long median epigastric incision and transected at the appropriate location depending on the tumor size. Moreover, intraoperative frozen sections were examined to ensure R0 resection when the distal margin of resection was uncertain.

The main steps of DTR include the following: the jejunum was transected approximately 20 cm below the Treitz ligament, a side‐to‐side anastomosis of the esophagus was performed, and the distal jejunum was transected intracorporeally with a linear stapler. Gastrojejunostomy (GJ) was performed 12 ~ 15 cm below the esophagojejunostomy (EJ) site with linear staplers. An overlap jejunojejunostomy (JJ) was performed between the proximal jejunum and the distal jejunum 40 cm below the EJ with a linear stapler. The common openings of the above three anastomoses were closed by knotless barbed absorbable sutures (V‐Loc™ 180) under laparoscopy.

**FIGURE 2:**
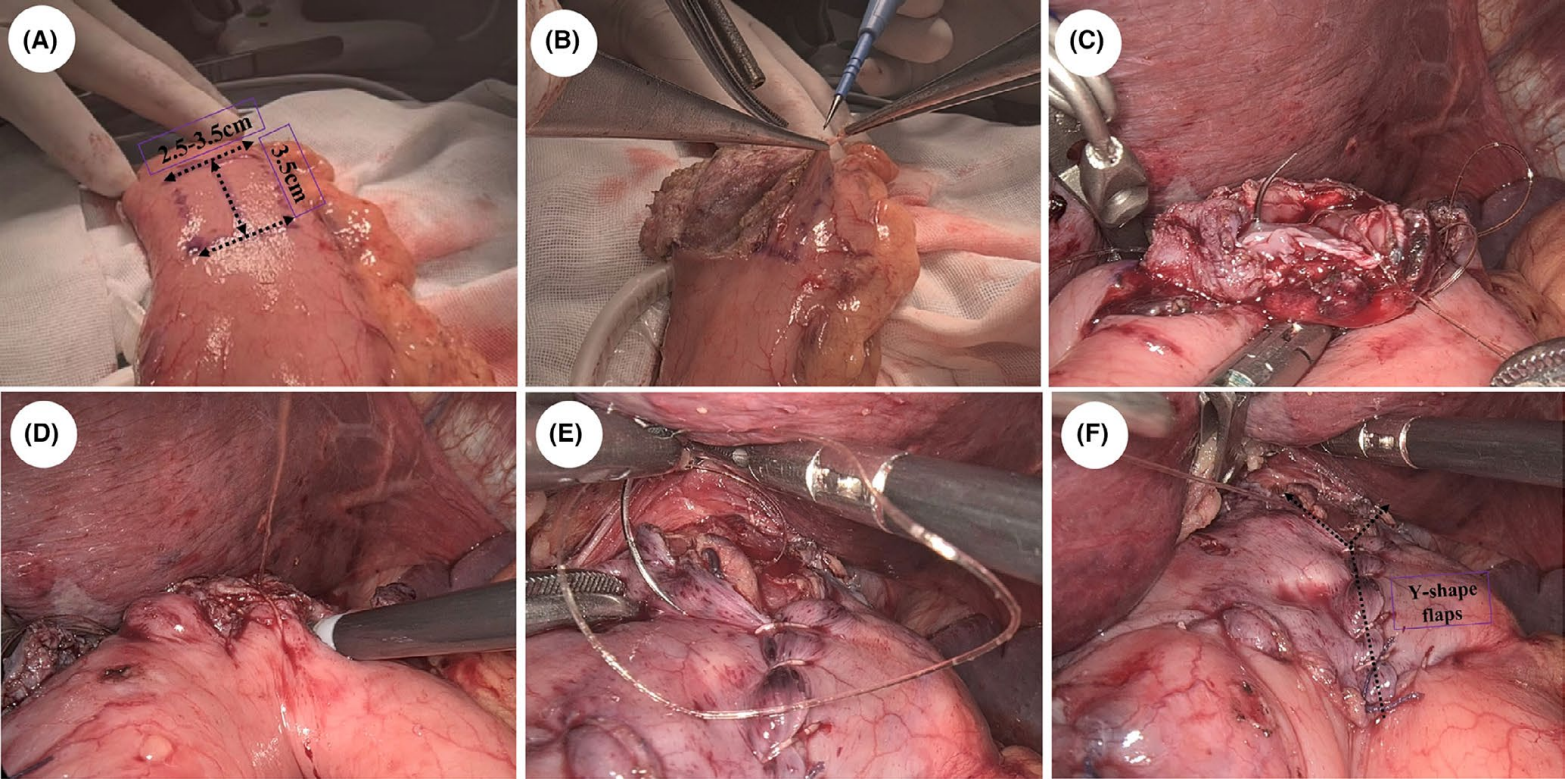
Esophagogastrostomy using the double flap technique. (A) “Double flap”, 2.5–3.5 cm × 3.5 cm (width × height). (B) Creation of the double seromuscular flaps is complete. (C) Continuous suturing between posterior wall of the esophagus and the inferior edge of the muco‐submucosal layer using V‐Loc™. (D) Continuous suturing between the anterior esophageal wall and the whole layer of the remnant stomach wall using V‐Loc™. (E) Continuous suturing between the anastomosis and the seromuscular flaps using V‐Loc™. (F) Y‐shape flaps covered the anastomosis.

**FIGURE 3:**
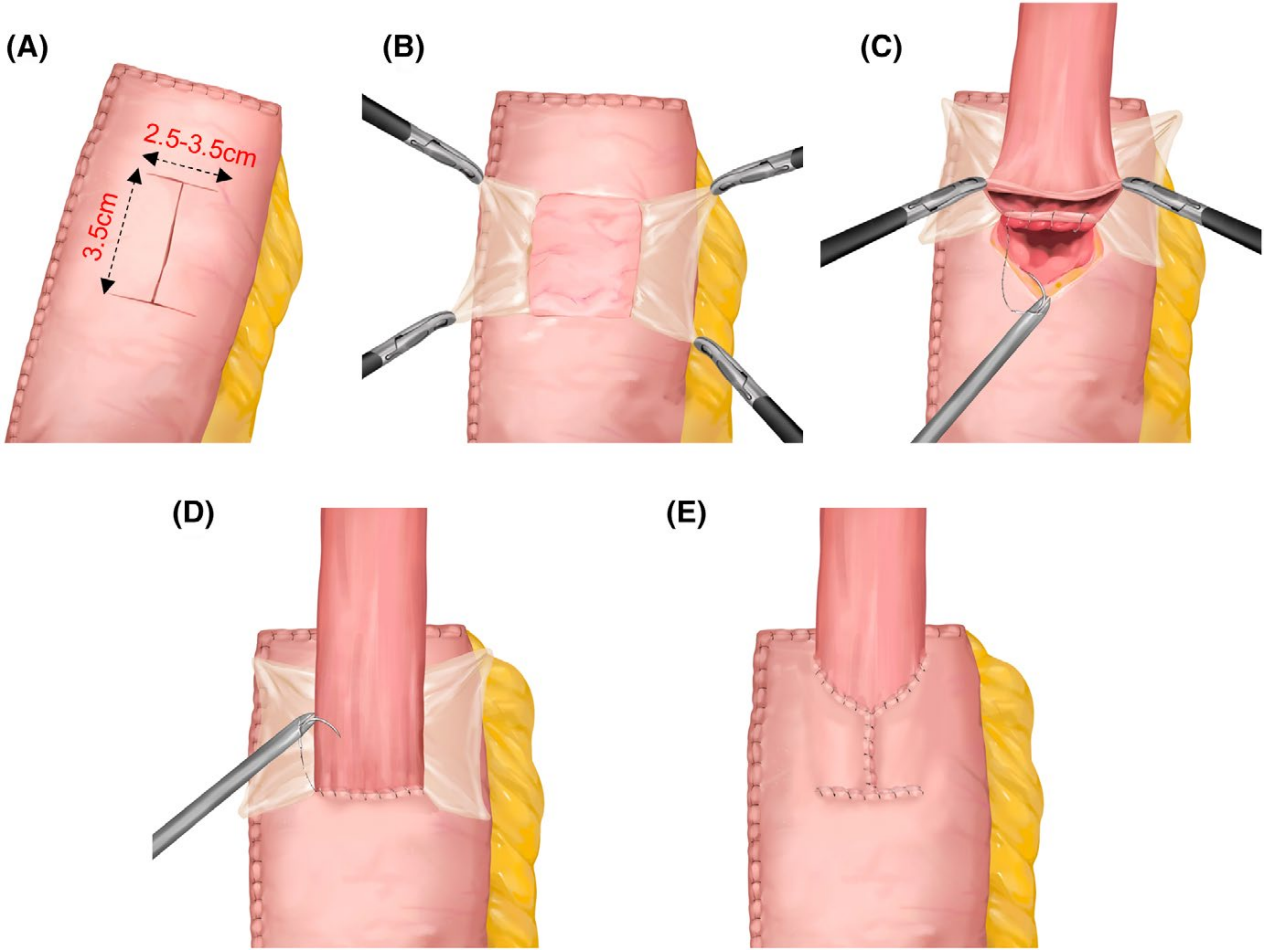
Schematic diagram of the double flap technique. (A) Complete the H‐shape mark. (B) Creation of the double seromuscular flaps. (C) Anastomosis of the posterior wall. (D) Anastomosis of the anterior wall. (E) Continuous suturing between the anastomosis and the seromuscular flaps.

In the DFT procedure, we made an H‐shaped (2.5–3.5 cm wide × 3.5 cm high) mark 3–4 cm below the margin of the anterior remnant stomach wall with methylene blue after transecting the proximal stomach extracorporeally (Figures 2A and 3A). Remarkably, the width of this H‐shaped marking was 2.5–3.5 cm, and the dimensions depended on the diameter of the esophagus evaluated by preoperative computed tomography or intraoperative exploration to establish the normal tension of the anastomosis. Double seromuscular flaps were created using electric cautery to carefully dissect the serosa and muscular layer along the H‐shaped marking, after which the submucosal layer was exposed (Figures 2B and 3B). Before the remnant stomach was returned to the abdominal cavity, the inferior edge of the muco‐submucosal window was opened in preparation for subsequent anastomosis.

The remaining steps were performed laparoscopically, and all sutures were hand‐sewn intracorporeally. Four sutures were used to secure the esophagus on the superior edge of the muco‐submucosal window at the previously marked points. Next, the esophageal stump was opened for esophagogastrostomy (EG). The posterior esophageal wall and the inferior edge of the muco‐submucosal layer were sutured continuously with V‐Loc™ 180 (Figures 2C and 3C), and anastomosis between the anterior esophageal wall and the whole layer of the anterior stomach wall was performed using the same strategy at the lower end of the window (Figures 2D and 3D). Finally, interrupted sutures were used to suture both side ends of the flaps together as a preliminary fix, and continuous suturing was performed between the flaps and the anastomosis using V‐Loc™ (Figure 2E). This procedure led to the anastomosis being completely wrapped and reinforced by the flaps (Figures 2F and 3E). It should be emphasized that DFT was not always performed intra‐abdominally. For a few patients with insufficient length of the abdominal segment of the esophagus, we attempted intra‐mediastinal DFT to explore its feasibility and efficacy and extreme caution should be exercised during operation to avoid damaging structures such as the pleura.

### 2.3 Clinical analysis and data collection

The background characteristics and postoperative results of all patients were obtained from the electronic medical records system. The clinicopathologic features included age, sex, BMI, ASA‐PS, Lauren classification, tumor size, pT stage, pN stage, pathological stage, preoperative comorbidities, and adjuvant chemotherapy. The surgical outcomes, including operation time, anastomosis time, estimated blood loss, and the number of retrieved LNs, were also recorded. The postoperative outcomes were days of gas‐passing, days of starting diet, postoperative length of hospital stay, and early (within 30 days of surgery) and late (after 30 days) complications of surgery. The Clavien–Dindo classification of surgical complications was used to classify the severity of postoperative complications.^19^ The follow‐up was conducted through the outpatient department, telephone visits, and internet communication, and the details included the following: (1) Body weight and hematologic parameters: total protein, albumin, hemoglobin, total cholesterol, vitamin B12, and lymphocyte counts. Moreover, the Controlling Nutritional Status (CONUT) score was calculated as a comprehensive indicator of nutritional status.^20^ (2) The results of endoscopy 1 year after surgery and reflux esophagitis were graded according to the Los Angeles Classification System.^21^ (3) The Postgastrectomy Syndrome Assessment Scale (PGSAS)‐45 was used to evaluate QOL.^22^

### 2.4 Statistical analysis

All statistical analyses were performed with SPSS 24.0 (SPSS/IBM). Continuous variables are presented as the mean ± standard deviation (SD) or median (range), and Student's *t* test or the Mann–Whitney *U* test was used for intergroup comparisons. Classification variables are expressed as a percentage (%), and Fisher's exact test was applied to compare differences between the two groups. We used a logistic regression model with a caliper of 0.2 standard deviations for PSM analysis. All P values cited were two‐sided, and *p* < 0.05 was considered to indicate statistical significance.

## 3 RESULTS

### 3.1 Patient characteristics

Table 1 details the clinicopathological characteristics of patients who underwent LPG‐DTR and LPG‐DFT before and after matching. Before matching, the two groups differed significantly in terms of age (*p* = 0.001), BMI (*p* = 0.040), and tumor size (*p* = 0.019). Regarding adjuvant chemotherapy, S‐1 and SOX were frequently adopted in patients with pathological stage II. After matching, there were no significant differences in clinicopathological characteristics between the DTR and DFT groups.

**TABLE 1:** Clinicopathological characteristics of the patients before and after PSM.

	Before matching			After matching		
	LPG with DTR (*n* = 80)	LPG with DFT (*n* = 24)	*p* Value	LPG with DTR (*n* = 48)	LPG with DFT (*n* = 24)	*p* Value
Age	67.5 ± 7.1	62.4 ± 5.2	0.001*	64.0 ± 5.3	62.4 ± 5.2	0.237
Sex			0.420			0.582
Male	60 (83.3%)	16 (66.7%)		35 (72.9%)	16 (66.7%)	
Female	20 (16.7%)	8 (33.3%)		13 (27.1%)	8 (33.3%)	
BMI (kg/m^2^)	22.1 ± 2.2	23.1 ± 2.0	0.040*	22.2 ± 2.0	23.1 ± 2.0	0.080
ASA‐PS			0.186			0.123
1	28 (35.0%)	4 (16.6%)		19 (39.6%)	4 (16.6%)	
2	43 (53.8%)	18 (75.0%)		24 (50.0%)	18 (75.0%)	
3	9 (11.2%)	2 (8.4%)		5 (10.4%)	2 (8.4%)	
Lauren classification			0.974			0.723
Intestinal	67 (83.8%)	20 (83.3%)		43 (89.6%)	20 (83.3%)	
Diffuse	9 (11.2%)	3 (12.5%)		3 (6.2%)	3 (12.5%)	
Mixed	4 (5.0%)	1 (4.2%)		2 (4.2%)	1 (4.2%)	
Histology type			0.933			0.699
Well differentiated	20 (25.0%)	7 (29.1%)		13 (27.1%)	7 (29.1%)	
Moderately differentiated	53 (66.3%)	15 (62.5%)		33 (68.7%)	15 (62.5%)	
Poorly differentiated	7 (8.7%)	2 (8.4%)		2 (4.2%)	2 (8.4%)	
Tumor size (cm)	2.0 ± 0.4	1.8 ± 0.5	0.019*	1.9 ± 0.4	1.8 ± 0.5	0.394
pT			0.368			0.340
T1a	12 (15.0%)	5 (20.8%)		6 (12.5%)	5 (20.8%)	
T1b	31 (38.7%)	5 (20.8%)		19 (39.6%)	5 (20.8%)	
T2	29 (33.3%)	12 (50.0%)		17 (35.4%)	12 (50.0%)	
T3	8 (10.0%)	2 (8.4%)		6 (12.5%)	2 (8.4%)	
pN			0.759			1.000
N0	65 (81.3%)	21 (87.5%)		41 (85.4%)	21 (87.5%)	
N1	15 (18.7%)	3 (12.5%)		7 (14.6%)	3 (12.5%)	
Pathologic stage			0.938			0.664
IA	38 (47.5%)	10 (41.5%)		25 (52.1%)	10 (41.5%)	
IB	28 (35.0%)	10 (41.6%)		13 (27.1%)	10 (41.6%)	
IIA	10 (12.5%)	3 (12.5%)		7 (14.6%)	3 (12.5%)	
IIB	4 (5.0%)	1 (4.2%)		3 (6.2%)	1 (4.2%)	
Preoperative comorbidity			0.542			0.737
Yes	39 (48.8%)	10 (41.6%)		22 (45.8%)	10 (41.6%)	
No	41 (51.2%)	14 (58.4%)		26 (54.2%)	14 (58.4%)	
Adjuvant chemotherapy			0.790			1.000
Yes	14 (17.5%)	3 (12.5%)		7 (14.6%)	3 (12.5%)	
No	66 (82.5%)	21 (87.5%)		41 (85.4%)	21 (87.5%)	

*Note*: The data are presented as mean ± standard deviation or n (%).

Abbreviations: ASA, American Society of Anesthesiologists physical status classification; BMI, body mass index; DFT, double flap technique; DTR, double‐tract reconstruction; LPG, laparoscopic proximal gastrectomy; PSM, propensity score matching.

* *p* < 0.05.

### 3.2 Surgical outcomes and postoperative complications

A comparison of surgical outcomes and postoperative complications between the two groups is shown in Table 2. The operative time, estimated blood loss, and number of retrieved LNs were comparable between the two groups. Nevertheless, the anastomosis time was significantly longer in the DFT group than in the DTR group (70.1 vs. 52.7 min, *p* < 0.001). In addition, the days of gas‐passing and the days of starting diet were shorter in the DFT group than in the DTR group (3.0 vs. 4.0 days, *p* < 0.001) and (4.0 vs. 5.0 days, *p* < 0.001), respectively. The postoperative length of hospital stay was significantly shorter in the DFT group (8.5 vs. 10.0 days, *p* < 0.001).

**TABLE 2:** Surgical outcomes and postoperative complications between the DTR and DFT groups.

	LPG with DTR (*n* = 48)	LPG with DFT (*n* = 24)	*p* Value
Operation time (min)	257.4 ± 41.2	277.7 ± 43.2	0.057
Anastomosis time (min)	52.7 ± 6.2	70.1 ± 6.9	<0.001*
Estimated blood loss (mL)	56.5 ± 11.3	62.1 ± 16.7	0.144
Number of retrieved LNs	24.2 ± 3.6	23.7 ± 3.8	0.539
Gas‐passing (days)	4.0 (3.0–6.0)	3.0 (2.0–4.0)	<0.001*
Start of diet (days)	5.0 (3.0–8.0)	4.0 (3.0–5.0)	<0.001*
Postoperative hospital stay (days)	10.0 (8.0–13.0)	8.5 (7.0–13.0)	<0.001*
Early complications	6 (12.5%)	2 (8.3%)	0.710
Fluid collection/Abscess	2 (4.2%)	0	0.549
Pneumonia	3 (6.3%)	2 (8.3%)	1.000
Intestinal obstruction	0	0	
Anastomotic leakage	1 (2.1%)	0	1.000
Anastomotic stenosis	0	0	
Bleeding	0	0	
Wound	1 (2.1%)	0	1.000
Postoperative mortality	0	0	
Late complications	5 (10.4%)	2 (8.3%)	1.000
Intestinal obstruction	1 (2.1%)	0	1.000
Internal hernia	0	0	
Cholecystitis	0	0	
Anastomotic leakage	0	0	
Anastomotic stenosis	1 (2.1%)	1 (4.2%)	1.000
Reflux esophagitis	3 (6.3%)	1 (4.2%)	1.000
≥Grade II C–D Score	3 (6.3%)	1 (4.2%)	1.000

*Note*: The data are presented as mean ± standard deviation, median (range) or *n* (%).

Abbreviations: C‐D, Clavien–Dindo classification of complication severity; DFT, double flap technique; DTR, double‐tract reconstruction; LNs, lymph nodes; LPG, laparoscopic proximal gastrectomy.

* *p* < 0.05.

Although the rates of early and late complications were lower in the DFT group (both 8.3%) than in the DTR group (12.5% and 10.4%), there were no significant differences between them (*p* = 0.710 and *p* = 1.000, respectively). No patients died in either group. Three patients in the DTR group and two patients in the DFT group experienced pneumonia, and all the patients were cured after active treatment with antibiotics. One patient in the DTR group underwent conservative treatment for small intestinal obstruction 3 months after surgery, and three patients developed reflux esophagitis (Grade B) 1 year after surgery and received antireflux drug therapy. Besides, only one patient in the DFT group developed reflux esophagitis (Grade B) without significant reflux related symptoms (Table S1 and Figure S1).

To clarify the potential impact of anastomotic sites in DFT on clinical outcomes, 24 patients in the DFT group were divided into two groups, intra‐abdominal DFT (IA‐DFT, *n* = 18) or intra‐mediastinal DFT (IM‐DFT, *n* = 6), and postoperative complications were further compared. However, as shown in Table S2, there were no significant differences for incidence of postoperative complications between the two groups.

### 3.3 Body weight and nutritional status

**FIGURE 4:**
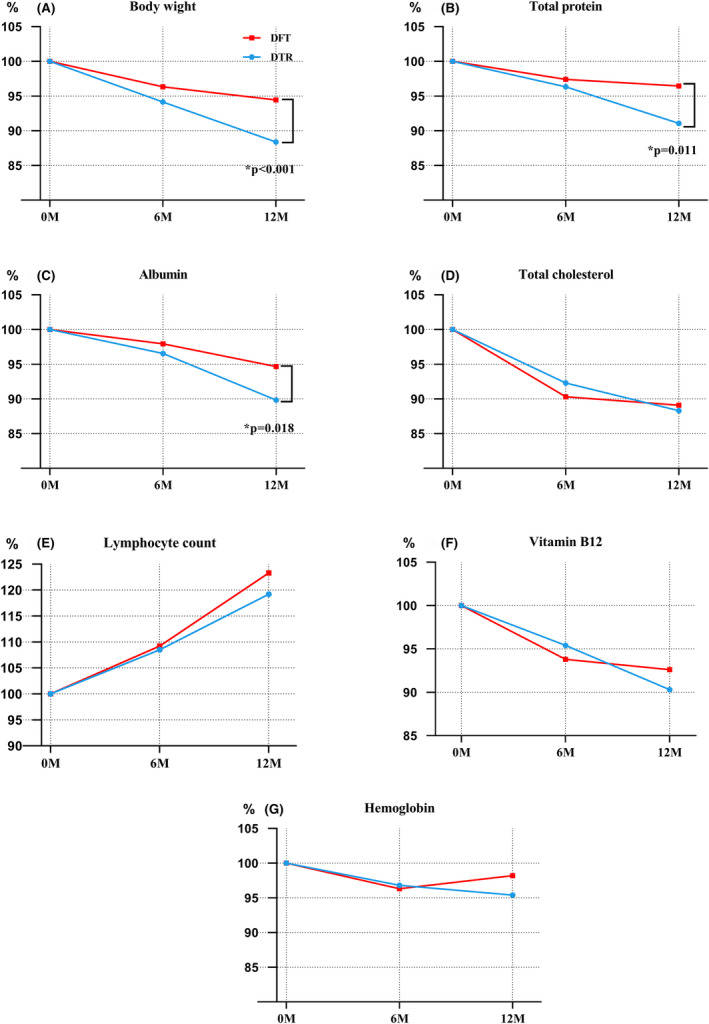
The rate of change in (A) body weight and nutritional status in terms of (B) total protein, (C) albumin, (D) total cholesterol, (E) lymphocyte count, (F) vitamin B12, and (G) hemoglobin between the DTR and DFT groups preoperatively and postoperatively at 6 and 12 months. DFT, double flap technique; DTR, double‐tract reconstruction. *p < 0.05.

Figure 4 shows the changes in body weight and hematologic parameters in the DTR and DFT groups. The rate of body weight loss in the DFT group was less than that in the DTR group at 6 and 12 months after surgery (Figure 4A) but was significantly different only at 12 months after surgery (−5.6% vs. −11.6%, *p* = 0.012). Similarly, postoperative total protein and albumin levels tended to decrease in both groups and were significantly higher in the DFT group than in the DTR group at 12 months after surgery (−3.5% vs. −9.9%, *p* = 0.011; −4.5% vs. −10.2%, *p* = 0.018, respectively; Figure 4B,C). There were no significant differences in the rates of change in total cholesterol, lymphocyte count, hemoglobin, or vitamin B12 between the DTR and DFT groups at any point after surgery (Figure 4D–G).

Furthermore, we calculated the CONUT score for both groups (Table S3). The preoperative scores of the two groups were comparable, and no patients experienced severe malnutrition (score 9–12) within 1 year after surgery. However, the CONUT score did not differ between the two groups at 6 or 12 months after surgery.

### 3.4 Postoperative QOL assessment

All questionnaires were taken back in the DTR and DFT groups, and the median duration of the QOL survey was 16 months after surgery. As summarized in Table 3, DTR showed better results in the meal‐related distress subscale (DTR 1.6 vs. DFT 2.1, *p* < 0.001). DFT was superior to DTR in terms of diarrhea subscale, constipation subscale and dumping subscale (DFT 1.6 vs. DTR 1.8, *p* = 0.005; DFT 1.8 vs. DTR 2.0, *p* = 0.022; DFT 1.4 vs. DTR 1.6, *p* = 0.007, respectively). However, the seven subscales did not influence the total symptom score (DTR 1.8 vs. DFT 1.7, *p* = 0.520), which indicates acceptable postoperative symptoms in both groups.

**TABLE 3:** Postoperative quality of life (QOL) assessment with PGSAS‐45.

	LPG with DTR (*n* = 48)	LPG with DFT(*n* = 24)	*p* Value
Symptoms			
*Esophageal reflux subscale*	1.3 ± 0.2	1.3 ± 0.3	0.570
*Abdominal pain subscale*	1.6 ± 0.3	1.7 ± 0.4	0.173
*Meal‐related distress subscale*	1.6 ± 0.5	2.1 ± 0.4	<0.001*
*Indigestion subscale*	2.4 ± 0.4	2.2 ± 0.4	0.112
*Diarrhea subscale*	1.8 ± 0.3	1.6 ± 0.2	0.005*
*Constipation subscale*	2.0 ± 0.3	1.8 ± 0.4	0.022*
*Dumping subscale*	1.6 ± 0.3	1.4 ± 0.2	0.007*
*Total symptom score*	1.8 ± 0.2	1.7 ± 0.1	0.520
Living status			
Ingested amount of food per meal^a^	6.3 ± 0.6	6.0 ± 0.6	0.038*
Necessity for additional meals	1.6 ± 0.5	1.9 ± 0.5	0.052
*Quality of ingestion subscale* ^a^	3.8 ± 0.5	3.8 ± 0.5	0.664
Ability for working	2.0 ± 0.5	2.0 ± 0.6	0.657
QOL			
Dissatisfaction with symptoms	1.8 ± 0.6	1.6 ± 0.7	0.274
Dissatisfaction at the meal	1.5 ± 0.5	1.5 ± 0.5	1.000
Dissatisfaction at working	1.7 ± 0.6	1.5 ± 0.5	0.258
*Dissatisfaction for daily life subscale*	1.7 ± 0.4	1.6 ± 0.3	0.209
*Physical component summary* ^a^	59.7 ± 5.6	62.5 ± 6.7	0.082
*Mental component summary* ^a^	58.9 ± 4.0	60.0 ± 5.2	0.564

*Note*: The data are presented as mean ± standard. Integrated subscales are italicized in the table. For outcome measures with ^a^ higher score indicates better condition; For other outcome measures, a higher score indicated a worse condition.

* *p* < 0.05.

## 4 DISCUSSION

Many studies have demonstrated oncologic safety and improved postoperative nutritional status after PG.^7^, ^8^, ^11^, ^14^, ^15^ Nevertheless, due to the variety of reconstruction methods, deciding which method can provide the most benefits remains a matter of controversy. The results of our study showed the advantages of DFT in several aspects.

Double tract reconstruction was first introduced by Aikou et al.^23^ As a modification of esophagogastrostomy (EG), DFT was first reported by Kamikawa et al.^24^ and both techniques have shown excellent results in reducing postoperative reflux esophagitis.^25^ A previous meta‐analysis comparing different reconstruction techniques after PG reported that the pooled incidence of reflux esophagitis for DFT was 8.9%, which was not significantly different from DTR (8.6%), but both incidences were significantly lower than that in EG (19.3%).^26^

**FIGURE 5:**
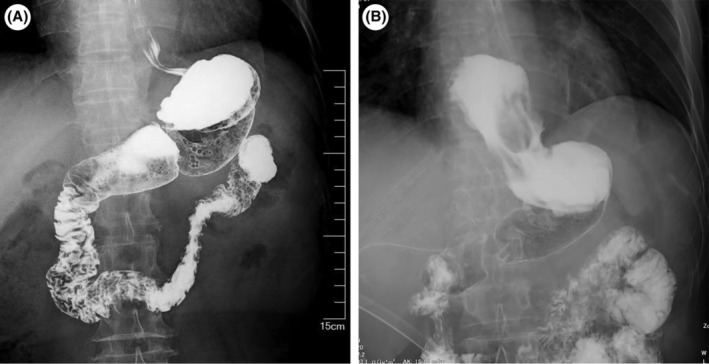
Upper gastrointestinal radiography for patients in the double flap technique group. (A) Intra‐abdominal double flap technique. (B) Intra‐mediastinal double flap technique (patients were at a position 30° head lower).

The length of the interposed jejunum between the EJ and GJ in the DTR may affect the prevention of reflux esophagitis, and the length was 10–15 cm in most studies.^12^, ^16^ Ma et al.^27^ reported that properly extending the length of the interposed jejunum to 15–20 cm according to the size of the remnant stomach could be more effective at preventing reflux esophagitis. However, it should be considered that the digestive tract torsion and difficulty of postoperative endoscopy are due to excessive length.^28^ Compared to DTR, DFT constructs a similar physiological digestive pathway, and its ingenious anastomotic design can preserve the functions of the excised cardiac and lower esophageal sphincters to a certain extent. Upper gastrointestinal radiography was routinely performed on postoperative day 7. In the DFT group, the contrast media did not regurgitate into the esophagus, even though the patients were at a position 30° head lower (Figure 5). The DFT appears to be the most promising antireflux procedure, yet the relatively high incidence of anastomotic stenosis after DFT is still problematic.

The incidence of anastomotic stenosis after DTR in previous studies ranged from 0 to 13.3%, and one patient (2.1%) from the DTR group in our cohort had anastomotic stenosis, which is consistent with the results of a previous study.^29^ Saze et al.^30^ reported that DFT was superior to other reconstruction methods for reducing reflux symptoms. In the DFT procedure, the tension at the anastomosis site is theoretically higher than that in other methods, which may cause postoperative anastomotic stenosis because the two seromuscular flaps are tightly wrapped around the lower esophagus, as reported in previous studies.^13^, ^14^, ^31^ The width of the H‐shaped seromuscular flaps is usually 2.5 cm, whereas we created the H‐shaped seromuscular flap with a width of 2.5–3.5 cm based on the diameter of the esophagus so that the compression from the seromuscular flaps could be undermined during the subsequent embedding of the esophagus.

Saeki et al.^32^ reported the safety and feasibility of V‐Loc in DFT. Hosoda et al.^33^ also suggested that V‐Loc was able to reduce the incidence of anastomotic stenosis in DFT. The present study similarly demonstrated the advantages of DFT because only one (4.2%) patient in the DFT group exhibited anastomotic stenosis. In addition, V‐Loc can greatly reduce the difficulty of hand‐sewing because no ligation is needed, and the anastomosis time can be relatively shortened. Therefore, we suggest that this modified DFT procedure can be performed by surgeons with extensive experience in minimally invasive surgery and that surgeons should pay more attention to completely aligning the tissues and controlling the stitch length to maintain stable tension in the anastomosis.

Regarding postoperative nutritional status, both groups showed varying degrees of reduced body weight and nutritional parameters compared to those observed during the preoperative period. However, the body weight and albumin levels at 1 year after surgery were higher in the DFT group than in the DTR group, similar to the results of a previous meta‐analysis,^17^ and these differences were related to the different digestive pathways of food. In DFT, all food enters the remnant stomach and passes through the duodenum, through which chyme can be mixed with pancreatic juice and gastrointestinal hormones. Compared to DFT, only a few foods enter the remnant stomach directly in DTR. Ahn et al.^12^ reported that the proportion of food entering the remnant stomach in DTR patients was approximately 60%. Wang et al.^34^ concluded that an appropriately enlarged gastrojejunal anastomosis in DTR patients would have advantages in terms of postoperative nutritional status compared with that in TG‐RY patients due to more food entering the duodenal pathway. These studies indicated that appropriately expanding the size of gastrojejunostomy in DTR may contribute to improving postoperative nutritional status.

Postoperative QOL after PG is also a great concern. Generally, heartburn and acid regurgitation caused by reflux esophagitis after PG severely impair QOL. In our study, the esophageal reflux subscale score of the DFT was as good as that of the DTR. In addition, we found that the results of the meal‐related distress subscale, which included the sense of food sticking, postprandial fullness, and early satiation, were worse in the DFT group. We speculate that the jejunum pathway in the DTR may help to facilitate the emptying of the remnant stomach to reduce the fullness. However, inadequate food digestion may be the main reason why patients in the DTR group experienced more intense diarrhea, constipation, and dumping. Kunisaki et al.^35^ completed a PGSAS‐45 NEXT study, which found that although PG is beneficial to improving QOL compared with TG, the main outcome measures of PGSAS‐45 could be easily affected by various background factors, such as age, gender, and adjuvant chemotherapy. Our study was limited by the number of patients and could not conduct relevant multivariate analysis to further clarify the advantages of DFT over DTR. However, the results of univariate analysis preliminarily suggest that DFT is a digestive tract reconstruction method after proximal gastrectomy worthy of promotion.

This study has several limitations. First, the retrospective nature of the study cannot be ignored. Although we used PSM to balance the baseline data, selection bias remained, and the patients ultimately included were limited and possibly underrepresented. Second, our center performed these two techniques relatively late, so we obtained only short‐term outcomes; however, follow‐up will continue to evaluate their long‐term efficacy. Third, for the assessment of postoperative nutritional status, we only compared preoperative, 6‐month, and 1‐year postoperative data, which may have resulted in unobserved differences between the two reconstruction methods at a certain postoperative stage.

In conclusion, this study revealed that despite the complexity of the procedure and longer anastomosis time, DFT emerged as a superior alternative to DTR in terms of facilitating early postoperative recovery, sustaining nutritional status, and improving QOL. DFT may be a promising procedure after PG. However, large‐sample, prospective, randomized trials should be conducted to validate these results.

## AUTHOR CONTRIBUTIONS

**Lindi Cai:** Writing – original draft. **Guanglin Qiu:** Writing – original draft. **Mengke Zhu:** Investigation; methodology; software. **Shangning Han:** Investigation; methodology; validation. **Pengwei Zhao:** Visualization. **Panxing Wang:** Investigation; visualization. **Xiaowen Li:** Investigation; validation. **Xinhua Liao:** Data curation. **Xiangming Che:** Data curation; project administration. **Lin Fan:** Project administration; writing – review and editing.

## FUNDING INFORMATION

This work was supported by grants from the Key Research and Development Projects of Shaanxi Province (2021SF‐123; 2023‐YBSF‐620; 2023‐YBSF‐624).

## CONFLICT OF INTEREST STATEMENT

The authors have no conflicts of interest to declare.

## ETHICS STATEMENT

Approval of the research protocol: This retrospective cohort study conformed to the provisions of the Declaration of Helsinki and was approved by the Ethics Committee of the First Affiliated Hospital of Xi'an Jiaotong University.

Informed Consent: Due to the retrospective design of this study, the requirement for informed consent was waived.

Registry and the registration no. of the study/trial: N/A.

Animal studies: N/A.

## Supporting information


Data S1:

## References

[1] Sung H , Ferlay J , Siegel RL , Laversanne M , Soerjomataram I , Jemal A , et al. Global cancer statistics 2020: GLOBOCAN estimates of incidence and mortality worldwide for 36 cancers in 185 countries. CA Cancer J Clin. 2021;71(3):209–249. 33538338 10.3322/caac.21660

[2] Ahn HS , Lee HJ , Yoo MW , Jeong SH , Park DJ , Kim HH , et al. Changes in clinicopathological features and survival after gastrectomy for gastric cancer over a 20‐year period. Br J Surg. 2011;98(2):255–260. 21082693 10.1002/bjs.7310

[3] Liu K , Yang K , Zhang W , Chen X , Chen X , Zhang B , et al. Changes of esophagogastric junctional adenocarcinoma and gastroesophageal reflux disease among surgical patients during 1988–2012: a single‐institution, high‐volume experience in China. Ann Surg. 2016;263(1):88–95. 25647058 10.1097/SLA.0000000000001148 PMC4679348

[4] Adachi Y , Kitano S , Sugimachi K . Surgery for gastric cancer: 10‐year experience worldwide. Gastric Cancer. 2001;4(4):166–174. 11846059 10.1007/s10120-001-8007-7

[5] Ooki A , Yamashita K , Kikuchi S , Sakuramoto S , Katada N , Hutawatari N , et al. Clinical significance of total gastrectomy for proximal gastric cancer. Anticancer Res. 2008;28(5B):2875–2883. 19031928

[6] Fujiya K , Kawamura T , Omae K , Makuuchi R , Irino T , Tokunaga M , et al. Impact of malnutrition after gastrectomy for gastric cancer on long‐term survival. Ann Surg Oncol. 2018;25(4):974–983. 10.1245/s10434-018-6342-8 29388124

[7] Yamasaki M , Takiguchi S , Omori T , Hirao M , Imamura H , Fujitani K , et al. Multicenter prospective trial of total gastrectomy versus proximal gastrectomy for upper third cT1 gastric cancer. Gastric Cancer. 2021;24(2):535–543. 33118118 10.1007/s10120-020-01129-6

[8] Ichikawa D , Komatsu S , Kubota T , Okamoto K , Shiozaki A , Fujiwara H , et al. Long‐term outcomes of patients who underwent limited proximal gastrectomy. Gastric Cancer. 2014;17(1):141–145. 23558459 10.1007/s10120-013-0257-7

[9] Takiguchi N , Takahashi M , Ikeda M , Inagawa S , Ueda S , Nobuoka T , et al. Long‐term quality‐of‐life comparison of total gastrectomy and proximal gastrectomy by postgastrectomy syndrome assessment scale (PGSAS‐45): a nationwide multi‐institutional study. Gastric Cancer. 2015;18(2):407–416. 24801198 10.1007/s10120-014-0377-8

[10] Shiraishi N , Hirose R , Morimoto A , Kawano K , Adachi Y , Kitano S . Gastric tube reconstruction prevented esophageal reflux after proximal gastrectomy. Gastric Cancer. 1998;1(1):78–79. 11957047 10.1007/s101209800023

[11] Nozaki I , Hato S , Kobatake T , Ohta K , Kubo Y , Kurita A . Long‐term outcome after proximal gastrectomy with jejunal interposition for gastric cancer compared with total gastrectomy. World J Surg. 2013;37(3):558–564. 23254949 10.1007/s00268-012-1894-4

[12] Ahn SH , Jung DH , Son SY , Lee CM , Park DJ , Kim HH . Laparoscopic double‐tract proximal gastrectomy for proximal early gastric cancer. Gastric Cancer. 2014;17(3):562–570. 24052482 10.1007/s10120-013-0303-5

[13] Kuroda S , Nishizaki M , Kikuchi S , Noma K , Tanabe S , Kagawa S , et al. Double‐flap technique as an antireflux procedure in esophagogastrostomy after proximal gastrectomy. J Am Coll Surg. 2016;223(2):e7–e13. 27157920 10.1016/j.jamcollsurg.2016.04.041

[14] Hayami M , Hiki N , Nunobe S , Mine S , Ohashi M , Kumagai K , et al. Clinical outcomes and evaluation of laparoscopic proximal gastrectomy with double‐flap technique for early gastric cancer in the upper third of the stomach. Ann Surg Oncol. 2017;24(6):1635–1642. 28130623 10.1245/s10434-017-5782-x

[15] Xiao JW , Liu ZL , Ye PC , Luo YJ , Fu ZM , Zou Q , et al. Clinical comparison of antrum‐preserving double tract reconstruction vs roux‐en‐Y reconstruction after gastrectomy for Siewert types II and III adenocarcinoma of the esophagogastric junction. World J Gastroenterol. 2015;21(34):9999–10007. 26379405 10.3748/wjg.v21.i34.9999 PMC4566393

[16] Yu B , Park KB , Park JY , Lee SS , Kwon OK , Chung HY , et al. Double tract reconstruction versus double flap technique: short‐term clinical outcomes after laparoscopic proximal gastrectomy for early gastric cancer. Surg Endosc. 2022;36(7):5243–5256. 34997340 10.1007/s00464-021-08902-3

[17] Huang QZ , Wang PC , Chen YX , Lin S , Ye K . Comparison of proximal gastrectomy with double‐flap technique and double‐tract reconstruction for proximal early gastric cancer: a meta‐analysis. Updates Surg. 2023;75(8):2117–2126. 37728858 10.1007/s13304-023-01638-w PMC10710383

[18] Japanese Gastric Cancer Association . Japanese gastric cancer treatment guidelines 2018. Gastric Cancer. 2021;24(1):1–21. 32060757 10.1007/s10120-020-01042-y PMC7790804

[19] Dindo D , Demartines N , Clavien PA . Classification of surgical complications: a new proposal with evaluation in a cohort of 6336 patients and results of a survey. Ann Surg. 2004;240(2):205–213. 15273542 10.1097/01.sla.0000133083.54934.ae PMC1360123

[20] Ignacio de Ulíbarri J , González‐Madroño A , de Villar NG , González P , González B , Mancha A , et al. CONUT: a tool for controlling nutritional status. First validation in a hospital population. Nutr Hosp. 2005;20(1):38–45. 15762418

[21] Lundell LR , Dent J , Bennett JR , Blum AL , Armstrong D , Galmiche JP , et al. Endoscopic assessment of oesophagitis: clinical and functional correlates and further validation of the Los Angeles classification. Gut. 1999;45(2):172–180. 10403727 10.1136/gut.45.2.172 PMC1727604

[22] Nakada K , Ikeda M , Takahashi M , Kinami S , Yoshida M , Uenosono Y , et al. Characteristics and clinical relevance of postgastrectomy syndrome assessment scale (PGSAS)‐45: newly developed integrated questionnaires for assessment of living status and quality of life in postgastrectomy patients. Gastric Cancer. 2015;18(1):147–158. 24515247 10.1007/s10120-014-0344-4

[23] Aikou T , Natsugoe S , Shimazu H , Nishi M . Antrum preserving double tract method for reconstruction following proximal gastrectomy. Jpn J Surg. 1988;18(1):114–115. 3386066 10.1007/BF02470857

[24] Kamikawa Y , Kobayashi T , Ueyama S , Kanbara K , Baba T , Fujii K . 噴 門側胃切除後の食道残胃吻合法における工夫ー徹底した逆流防止と安全 性を目指してー. Oper Dent. 1998;52:1477–1483.

[25] Nishimura E , Irino T , Matsuda S , Fukuda K , Nakamura R , Kawakubo H , et al. Comparison of changes in body‐fat mass and reflux esophagitis among reconstruction methods for proximal gastrectomy. Asian J Surg. 2023;46(1):394–398. 35570106 10.1016/j.asjsur.2022.04.110

[26] Shaibu Z , Chen Z , Mzee S , Theophilus A , Danbala IA . Effects of reconstruction techniques after proximal gastrectomy: a systematic review and meta‐analysis. World J Surg Oncol. 2020;18(1):171. 32677956 10.1186/s12957-020-01936-2 PMC7367236

[27] Ma X , Zhao M , Wang J , Pan H , Wu J , Xing C . Clinical comparison of proximal gastrectomy with double‐tract reconstruction versus total gastrectomy with roux‐en‐Y anastomosis for Siewert type II/III adenocarcinoma of the esophagogastric junction. J Gastric Cancer. 2022;22(3):220–234. 35938368 10.5230/jgc.2022.22.e25 PMC9359881

[28] Tokunaga M , Ohyama S , Hiki N , Hoshino E , Nunobe S , Fukunaga T , et al. Endoscopic evaluation of reflux esophagitis after proximal gastrectomy: comparison between esophagogastric anastomosis and jejunal interposition. World J Surg. 2008;32(7):1473–1477. 18264827 10.1007/s00268-007-9459-7

[29] Lu S , Ma F , Zhang Z , Peng L , Yang W , Chai J , et al. Various kinds of functional digestive tract reconstruction methods after proximal gastrectomy. Front Oncol. 2021;11:685717. 34414108 10.3389/fonc.2021.685717 PMC8369505

[30] Saze Z , Kase K , Nakano H , Yamauchi N , Kaneta A , Watanabe Y , et al. Functional benefits of the double flap technique after proximal gastrectomy for gastric cancer. BMC Surg. 2021;21(1):392. 34740344 10.1186/s12893-021-01390-1 PMC8569978

[31] Kuroda S , Choda Y , Otsuka S , Ueyama S , Tanaka N , Muraoka A , et al. Multicenter retrospective study to evaluate the efficacy and safety of the double‐flap technique as antireflux esophagogastrostomy after proximal gastrectomy (rD‐FLAP study). Ann Gastroenterol Surg. 2019;3(1):96–103. 30697614 10.1002/ags3.12216 PMC6345660

[32] Saeki Y , Tanabe K , Yamamoto Y , Ohta H , Saito R , Ohdan H . Laparoscopic proximal gastrectomy with hinged double flap method using knotless barbed absorbable sutures: a case series. Int J Surg Case Rep. 2018;51:165–169. 30172056 10.1016/j.ijscr.2018.08.041 PMC6122145

[33] Hosoda K , Washio M , Mieno H , Moriya H , Ema A , Ushiku H , et al. Comparison of double‐flap and OrVil techniques of laparoscopy‐assisted proximal gastrectomy in preventing gastroesophageal reflux: a retrospective cohort study. Langenbecks Arch Surg. 2019;404(1):81–91. 30612151 10.1007/s00423-018-1743-5

[34] Wang L , Xia Y , Jiang T , Li F , Wang W , Zhang D , et al. Short‐term surgical outcomes of laparoscopic proximal gastrectomy with double‐tract reconstruction versus laparoscopic Total gastrectomy for adenocarcinoma of esophagogastric junction: a matched‐cohort study. J Surg Res. 2020;246:292–299. 31630013 10.1016/j.jss.2019.09.022

[35] Kunisaki C , Yoshida K , Yoshida M , Matsumoto S , Arigami T , Sugiyama Y , et al. Effects of proximal gastrectomy and various clinical factors on postoperative quality of life for upper‐third gastric cancer assessed using the postgastrectomy syndrome assessment Scale‐45 (PGSAS‐45): a PGSAS NEXT study. Ann Surg Oncol. 2022;29(6):3899–3908. 34988838 10.1245/s10434-021-11136-1

