# Supplementary material for: Digestive tract reconstruction after laparoscopic proximal gastrectomy: Double tract reconstruction or double flap technique?
Source: Ann Gastroenterol Surg. 2024 Sep 1;9(1):98–108. doi: 10.1002/ags3.12857 (PMC11693543; doi:10.1002/ags3.12857)
Supplement: Supplementary file 1 — Data S1: [file AGS3-9-98-s001.docx]

Supplementary Table 1. Endoscopic findings 12 months after proximal gastrectomy.

| Reflux esophagitis  (Los Angeles classification) | LPG with DTR(*n*=48) | LPG with DFT(*n*=24) | *p* value |
| --- | --- | --- | --- |
| Grade A | 0 | 0 | - |
| Grade B | 3 (6.3%) | 1 (4.2%) | 1.000 |
| Grade C | 0 | 0 | - |
| Grade D | 0 | 0 | - |

The data are presented as n (%)

LPG, laparoscopic proximal gastrectomy; DTR, double-tract reconstruction; DFT, double flap technique.

Supplementary Table 2. Postoperative complications between the IA-DFT and IM- DFT groups.

|  | IA-DFT(*n*=18) | IM- DFT(*n*=6) | *p* value |
| --- | --- | --- | --- |
| **Early complications** | 1 (5.6%) | 1 (16.7%) | 0.446 |
| Fluid collection/Abscess | 0 | 0 |  |
| Pneumonia | 1 (5.6%) | 1 (16.7%) | 0.446 |
| Intestinal obstruction | 0 | 0 |  |
| Anastomotic leakage | 0 | 0 |  |
| Anastomotic stenosis | 0 | 0 |  |
| Bleeding | 0 | 0 |  |
| Wound | 0 | 0 |  |
| **Postoperative mortality** | 0 | 0 |  |
| **Late complications** | 2 (11.2%) | 0 | 1.000 |
| Intestinal obstruction | 0 | 0 |  |
| Internal hernia | 0 | 0 |  |
| Cholecystitis | 0 | 0 |  |
| Anastomotic leakage | 0 | 0 |  |
| Anastomotic stenosis | 1 (5.6%) | 0 | 1.000 |
| Reflux esophagitis | 1 (5.6%) | 0 | 1.000 |
| **≥Grade II C–D Score** | 1 (5.6%) | 0 | 1.000 |

The data are presented as n (%)

IA-DFT, intra-abdominal double flap technique; IM-DFT, intra-mediastinal double flap technique; C-D, Clavien-Dindo classification of complication severity.

Supplementary Table 3. CONUT score between the DTR and DFT groups.

| CONUT score | LPG with DTR(*n*=48) | | | | LPG with DFT(*n*=24) | | | | *p* value |
| --- | --- | --- | --- | --- | --- | --- | --- | --- | --- |
|  | 0-1 | 2-4 | 5-8 | 9-12 | 0-1 | 2-4 | 5-8 | 9-12 |  |
| 0M | 13 (27.1%) | 29 (60.4%) | 6 (12.5%) | - | 8 (33.3%) | 16 (66.7%) | - | - | 0.211 |
| 6M | 9 (18.8%) | 30 (62.4%) | 9 (18.8%) | - | 8 (33.3%) | 13 (54.2%) | 3 (12.5%) | - | 0.298 |
| 12M | 9 (18.8%) | 28 (58.3%) | 11 (22.9%) | - | 6 (25.0%) | 15 (62.5%) | 3 (12.5%) | - | 0.576 |

The data are presented as n (%)

The Controlling Nutritional Status (CONUT) score, which includes measures of serum albumin, total cholesterol, and peripheral lymphocyte count. The nutritional Status of patients are divided into four categories according to CONUT score as follows: normal (score 0–1), light (score 2–4), moderate (score 5–8), and severe (score 9–12)

Supplementary Figure 1


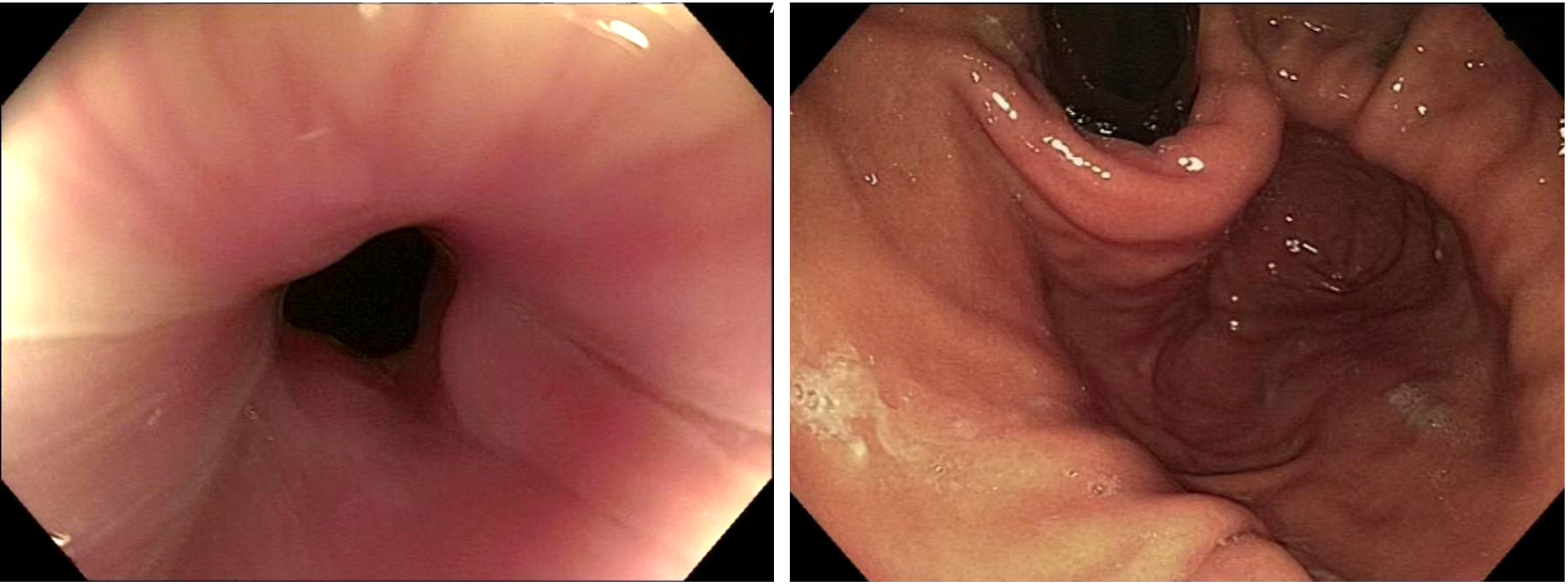


**Supplementary Figure 1** Representative endoscopic images 12 months after surgery in the DFT group.
